# Advantages of a Virtual Collaborative Research Dermatology Laboratory

**DOI:** 10.2196/65697

**Published:** 2025-10-30

**Authors:** Natasha E Barton, Kenny Ta, Angela R Loczi-Storm, Cory A Dunnick, Robert P Dellavalle

**Affiliations:** 1 School of Medicine, University of Colorado, 13001 E 17th Pl, Aurora, CO, 80045, United States, 1 3039604491; 2 School of Medicine, University of Minnesota, Minneapolis, MN, United States; 3 College of Osteopathic Medicine of the Pacific-Northwest, Western University of Health Sciences, Lebanon, OR, United States; 4 Department of Dermatology, University of Colorado, Aurora, CO, United States; 5 Department of Dermatology, University of Minnesota, Minneapolis, MN, United States

**Keywords:** medical students, collaborative research, virtual collaboration, dermatology research, tutorial, editorial

## Abstract

The Dellavalle/Dunnick Dermato-Epidemiology Lab transitioned from a single campus to a dual-campus collaboration between the University of Colorado and the University of Minnesota in 2024. Since the 2020 COVID-19 pandemic, the laboratory has been operating on Zoom and allows medical students from any institution to join. This innovative laboratory structure offers students and other researchers unique opportunities to engage in dermatological research and develop professional networks across two large academic institutions. The laboratory’s model embraces a virtual collaborative approach, promotes inclusivity, encourages student-led inquiry, and provides a structured environment for professional development and academic output. Through its commitment to diverse student perspectives and interdisciplinary cooperation, the Dellavalle/Dunnick Dermato-Epidemiology Lab creates a new, equitable, nationwide model for research and mentorship in dermatology, supporting medical students, residents, and fellows to navigate future careers in dermatology.

## Introduction

The Dellavalle/Dunnick Dermato-Epidemiology Lab represents an innovative collaboration between the University of Colorado (CU) and the University of Minnesota (UMN). This dual-campus model offers a unique platform for medical students, residents, and fellows, particularly those from institutions without dedicated dermatology programs, to engage in high-impact dermatological research. The laboratory’s goals are multifaceted: to foster professional networks, promote a culture of scientific curiosity and inquiry within the dermatology field, and enhance students’ research credentials. This editorial will summarize the key components and operational structure contributing to the laboratory’s sustained engagement and productivity across institutions, providing a practical framework for medical educators, researchers, or administrators looking to replicate this scalable model in other medical specialties. By focusing on strategies for creating an inclusive, collaborative research environment, we will explore how mentorship strategies, logistical planning, digital tools, and virtual collaboration can bridge gaps in medical education and support the next generation of physician-scientists, particularly in settings where formal dermatology programs or research infrastructure may be limited.

## Laboratory Background

The Dellavalle/Dunnick Dermato-Epidemiology Lab was founded as an in-person research group at CU. It operated under a traditional model of weekly on-site meetings, primarily with CU medical students. This structure had been in place since the laboratory’s creation in the early 2000s.

During the COVID-19 pandemic, the laboratory transitioned to a fully virtual format using platforms such as Zoom (Zoom Communications) and Google Workspace. This transition significantly expanded the laboratory’s geographic reach and accessibility, allowing medical students from over 30 institutions nationwide to participate. Participation is open and nonselective; students are not required to apply but are vetted and invited to join meetings and become involved as their interest develops.

While the laboratory has an accessible website, its growth has largely been driven by its strong reputation, word-of-mouth referrals, and visibility at academic conferences. The research credentials of the laboratory’s principal investigators, authors RPD and CAD, further enhance its appeal, attracting motivated trainees eager to contribute to impactful dermatological projects. RPD has an h-index of 70 and more than 145,000 citations, and CAD has an h-index of 36 and more than 5,000 citations [1,2].

When RPD transitioned to UMN, the lab’s commitment to virtual collaboration ensured continuity. Weekly Zoom meetings provide a platform for updates, allowing for the seamless integration of students and residents from multiple locations. This virtual approach supported the laboratory’s ongoing success, allowing it to thrive as a dual-campus operation.

The laboratory’s focus on collaboration has led to contributions from students at a range of institutions, including Rocky Vista University College of Osteopathic Medicine, Case Western Reserve University, Kansas City University, Texas Tech El Paso, A.T. Still University, SUNY Upstate University, Texas A&M, The Ohio State University, and many others. These efforts have resulted in numerous publications and sustained high-impact research projects, further cementing the laboratory’s role in advancing dermatological science through interdisciplinary collaboration.

## Laboratory Operations

### Joining the Laboratory

The Dellavalle/Dunnick Dermato-Epidemiology Lab uses an open-access, inclusive model for student participation. When students learn about the laboratory through the website, a conference presentation, or word of mouth, they email CAD and/or RPD. One of the unique aspects of our laboratory is that students are not required to apply or be selected to join. Instead, interested students are provided the Zoom link and the shared laboratory Google Doc (Textbox 1 and Multimedia Appendix 1). They are invited to attend weekly meetings without expectations of prior research experience.

Textbox 1. **Fictitious lab executive summary. Details included were created for the purposes of this tutorial and are not accurate or representative. Formatting is the same as the real shared document.**

**Dellavalle/Dunnick Dermato-Epidemiology Weekly Research Lab Meeting**

Time: Tuesdays 12:15 pm Mountain time zone (Denver)

Zoom: [Zoom Link]

Zoom meeting ID: 111 222 3333

Google doc: [Google Document Link]

Lab website: derm-epi.com

Dermatology Faculty:

Robert Dellavalle, MD, PhD, MSPH, 720-111-3333

Cory A. Dunnick, MD, 303-111-3333,

**Clinical Research Fellow, Lab Coordinators, Residents, Fellows:**

John Meisenheimer, MD

**U. Minnesota Medical Students:**

(2025)

(2026)

(2027): Kenny Ta

(2028)

**CU Medical Students:**

(2025)

(2026)

(2027): Natasha Barton

(2028)

**Medical Students at Other Institutions:**

Angela Loca-Storm (WesternU-COMPNW)

**Manuscripts Submitted:**

1. Advantages of a Virtual Collaborative Research Dermatology Laboratory - Natasha, Kenny, Angela, tutorial to *JMIR Med Education*

**Manuscripts Needing Revision:**

1. Advantages of a Virtual Collaborative Research Dermatology Laboratory - Natasha, Kenny, Angela, tutorial to *JMIR Med Education*

**Dormant Projects:**

1. Advantages of a Virtual Collaborative Research Dermatology Laboratory - Natasha, Kenny, Angela, tutorial to *JMIR Med Education*

**Active IRBs:**

1. COMIRB 111: Survey on Advantages of a Virtual Collaborative Research Dermatology Laboratory - Natasha, Kenny, Angela

**Active Grants:**

1. Survey on Advantages of a Virtual Collaborative Research Dermatology Laboratory - Natasha, Kenny, Angela

**Grants Available:**

1. Sulzberger Education AAD innovation grant (LOI opens June 2025 – $5K/30K)

**Upcoming Events:**

1. May 7-10, 2025, San Diego, SID
2. July 10-13, 2025, Chicago, AAD Innovation Academy
3. March 27-31, 2026 Denver, AAD
4. May 13-16, 2026 Chicago, SID

**Recommended:**

1. Brief Faculty Development Videos
2. Listen to Dermasphere blog (Spotify)

New participants are encouraged to observe their first few meetings to gain familiarity with the laboratory’s structure and active projects. As they become more comfortable, they are encouraged to contact established laboratory members to join projects that interest them. This approach reduces barriers to participation and allows self-directed engagement based on availability, interest, and experience.

### Weekly Operations

Consistent, weekly Zoom meetings serve as the backbone of laboratory operations. The meetings follow a structured format, including a “popcorn-style” check-in, where each member introduces themselves, their role, medical school, and current location. After the check-in, we engage in personal development activities, such as discussing key insights from recent conferences or watching brief faculty development videos from the Association of Professors of Dermatology [3].

Following the professional development segment, we review the current list of ongoing projects. This includes updates on submitted projects awaiting decisions, projects needing revisions, and new ideas. Laboratory members provide updates on their respective projects, and if papers or posters need review, the members will share their screen to go through the material with the group. We then go over any upcoming deadlines for conferences or grant applications. The meeting ends with a final “popcorn-style” check-out with the opportunity to provide any last-minute updates.

### Shared Laboratory Document

The shared laboratory document is key to our operations and is essentially the “holy grail” of the laboratory. This Google Doc is a centralized hub for all essential information related to laboratory activities. It includes a comprehensive list of laboratory members’ contact details, ensuring easy communication across our collaborative network.

Key sections of the document include the following:

- Lab member information
- Projects submitted awaiting decision (this section tracks all projects that have been submitted for review and are waiting for feedback or approval)
- Projects with revisions (this lists projects that have received reviewer comments and are currently undergoing revisions)
- Active projects (this section highlights ongoing research projects)
- Institutional review board (IRB) protocols (a list of all active IRB protocols currently in effect for our research projects)
- Active grants (this section includes information on approved grants and details on who is leading or contributing to each)
- Open conferences with submission deadlines (this section provides an updated list of conferences open for submissions, along with their deadlines)
- Grant submission deadlines (a list of upcoming grant applications with specific submission dates)
- Upcoming dermatology events (a calendar of dermatology-related events that may interest lab members for networking or continuing education)

The shared laboratory document lists the names of the members currently working on each active project, IRB protocol, and grant. This ensures that all laboratory members can stay updated on who is involved in each initiative and fosters a collaborative, transparent research environment. The document is updated weekly to ensure all members can access current information and deadlines.

### Project Development

Participation in the laboratory is self-motivated, and each member is encouraged to develop their own unique research ideas. Once an idea is proposed, our principal investigators, along with other laboratory members, offer suggestions and support to help get the project started. In addition to individual project ideas, our principal investigators often provide project concepts and assemble teams to work on them. After a project is initiated and a team is formed, smaller, more focused meetings take place outside of the weekly laboratory meetings to continue project work.

### Funding

The laboratory operates without dedicated, laboratory-specific funding. Instead, students are encouraged to seek financial support for their research projects through grants available at their home institutions or from national sources. While many projects do not require funding beyond literature access and student time, grant opportunities are essential for supporting conference travel, publication fees, and community outreach efforts. Students who secure funding are encouraged to share their grant proposals with other laboratory members as templates, creating a cycle of shared learning and resource building. The shared Google Doc also lists all active grants that laboratory members have received, as well as open grant applications, providing students with up-to-date opportunities to apply for funding. By using this collaborative approach to funding, the laboratory fosters a culture of self-sufficiency and resourcefulness, allowing students to secure financial support for their community-based research endeavors.

## Steps for Creating a Successful Collaborative Laboratory

### Step 1: Setting Clear Goals and Objectives for Research Programs

Every successful research program begins with a clear set of objectives that guide its mission and outcomes. The Dellavalle/Dunnick Dermato-Epidemiology Lab operates with well-defined goals rooted in the desire to offer students substantial research opportunities and mentorship while promoting the exploration of novel scientific questions. Key goals include providing professional mentorship, fostering a collaborative research environment that encourages scientific curiosity, and enhancing student research opportunities. These objectives are not only important for the success of the laboratory but are also critical for helping medical students navigate the competitive landscape of dermatology residency applications, which became more difficult with the transition of the United States Medical Licensing Examination Step I exam to pass/fail grading in 2022 [4,5].

To replicate this model, laboratory groups must first identify their core objectives. These could include goals such as improving students’ exposure to field-specific research, encouraging scientific curiosity, providing mentorship opportunities, and offering access to high-impact, publishable projects. Once these objectives are established, they will serve as a framework for the program’s success and provide clarity on how to best serve the students involved.

### Step 2: Structuring the Program for Success

The success of the Dellavalle/Dunnick Dermato-Epidemiology Lab is heavily dependent on its strong organizational structure and clear communication channels. To maintain the flow of projects and ensure consistent progress, the laboratory holds weekly meetings where students and faculty members provide updates on ongoing research, discuss any challenges, and plan the next steps. These regular check-ins foster a sense of continuity and accountability, which is especially important when managing research teams spread across multiple campuses. One of the key benefits of this structure is that it allows students from various institutions to remain engaged, regardless of their geographic location.

Weekly meetings and centralized documentation keep research organized and moving forward. Using tools like Google Docs supports team transparency and accountability. Replicating this model involves consistent communication, flexibility, and logistical coordination across institutions. In addition, it’s important to maintain a flexible, adaptable approach to account for the varying schedules and needs of participants from different institutions.

### Step 3: Creating an Inclusive and Collaborative Research Environment

The laboratory’s success can be attributed to its focus on creating a collaborative environment where students feel valued and empowered to contribute to meaningful research, regardless of their academic background. This ethos is reflected in the laboratory’s recruitment policy, which invites students from institutions with and without established dermatology programs to participate in research. This inclusive approach fosters camaraderie between MD and DO students, enhancing professional networks and building connections early in their careers.

Replicating such an inclusive environment requires institutions to recruit and include participants consciously and to create a culture that values all contributions, regardless of a student’s institutional affiliation. Offering mentorship and guidance from experienced researchers and ensuring that students from various backgrounds have equal access to resources and support are essential steps in creating a thriving research community.

### Step 4: Ensuring Program Sustainability and Resources

Long-term sustainability of a virtual collaborative research program requires strategic planning, flexible leadership, and efficient resource management. Many collaborative research programs rely on extramural grants and institutional support to finance operations. Program leaders should prioritize securing long-term funding sources, including government grants, philanthropic support, or institutional backing, to ensure the sustainability of the research program. Since most academic institutions already provide access to platforms like Zoom and Microsoft 365, investing in a solid technology infrastructure can significantly streamline communication and enhance collaboration.

### Step 5: Replicating the Model: Key Insights

To replicate the success of the Dellavalle/Dunnick Dermato-Epidemiology Lab, it’s important to focus on several core principles, outlined in Textbox 2.

Textbox 2. **Core principles.**

**Defining clear objectives**

Establish clear and measurable goals for the program, ensuring they align with students’ goals and the broader goals of your institution or research community.

**Structuring for organization**

Implement a consistent structure for regular meetings, project tracking, and task management.

**Promoting inclusivity**

Create an inclusive research environment that invites participation from students across multiple institutions and backgrounds and encourages collaboration.

**Securing resources**

Plan for the sustainability of the program by securing funding and investing in technology platforms that facilitate virtual collaboration.

**Performing ongoing evaluation**

Continuously assess the program’s impact and refine based on participant feedback.

## Conclusion

The Dellavalle/Dunnick Dermato-Epidemiology Lab offers a practical model for building scalable, inclusive, and collaborative multicampus research programs. Its structured virtual environment lowers access barriers, supports consistent mentorship, and engages students from schools without dermatology departments, addressing common gaps in academic medical training.

As medical education adapts to more decentralized and technology-driven formats, this model illustrates how virtual infrastructure and intentional design can sustain student scholarship and faculty engagement. By applying the principles outlined in this editorial—clear objectives, open access, consistent workflows, and distributed leadership—other institutions can replicate the success of the Dellavalle/Dunnick Dermato-Epidemiology Lab and contribute to the next generation of physician-scientists who are prepared to excel in an increasingly competitive medical landscape.

## Supplementary material

10.2196/65697 Multimedia Appendix 1 Fictitious laboratory executive summary. Details included were created for the purposes of this tutorial and are not accurate or representative. Formatting is the same as the real shared document.

## Abbreviations

CU: University of Colorado
IRB: institutional review board
UMN: University of Minnesota
