# Supplementary material for: Advantages of a Virtual Collaborative Research Dermatology Laboratory
Source: JMIR Med Educ. 2025 Oct 30;11:e65697. doi: 10.2196/65697 (PMC12574937; doi:10.2196/65697)
Supplement: Multimedia Appendix 1 [file mededu-v11-e65697-s001.docx]

**Dellavalle/Dunnick Dermato-Epidemiology Weekly Research Lab Meeting**

Time: Tuesdays 12:15 pm Mountain time zone (Denver)

Zoom: Zoom Link

Zoom meeting ID: 111 222 3333

Google doc: Google Document Link

Lab website**:** [derm-epi.com](http://derm-epi.com)

**Dermatology Faculty:**

Robert Dellavalle, MD, PhD, MSPH, 720-111-3333, email[@](mailto:della056@umn.edu)gmail.com

Cory A. Dunnick, MD, 303-111-3333, [cory.dunnick@va.gov](mailto:cory.dunnick@va.gov)

**Clinical Research Fellow, Lab Coordinators, Residents, Fellows:**

John Meisenheimer, MD [jmeisenh@umn.edu](mailto:jmeisenh@umn.edu)

**U. Minnesota Medical Students:**

(2025)

(2026)

(2027): Kenny Ta [ta000030@umn.edu](mailto:ta000030@umn.edu)

(2028)

**CU Medical Students:**

(2025)

(2026)

(2027): Natasha Barton [natasha.barton@cuanschutz.edu](mailto:natasha.barton@cuanschutz.edu)

(2028)

**Medical Students at Other Institutions:**

Angela Loczi-Storm [angela.loczistorm@westernu.edu](mailto:angela.loczistorm@westernu.edu) (WesternU-COMPNW)

**Manuscripts Submitted:**

1. Advantages of a Virtual Collaborative Research Dermatology Laboratory - Natasha, Kenny, Angela, tutorial to *JMIR Med Education*

**Manuscripts Needing Revision:**

1. Advantages of a Virtual Collaborative Research Dermatology Laboratory - Natasha, Kenny, Angela, tutorial to *JMIR Med Education*

**Manuscripts in Preparation:**

1. Advantages of a Virtual Collaborative Research Dermatology Laboratory - Natasha, Kenny, Angela, tutorial to *JMIR Med Education*

**Dormant Projects:**

1. Advantages of a Virtual Collaborative Research Dermatology Laboratory - Natasha, Kenny, Angela, tutorial to *JMIR Med Education*

**Active IRBs:**

1. COMIRB 111: Survey on Advantages of a Virtual Collaborative Research Dermatology Laboratory - Natasha, Kenny, Angela

**Active Grants:**

1. Survey on Advantages of a Virtual Collaborative Research Dermatology Laboratory - Natasha, Kenny, Angela

**Grants Available:**

1. Sulzberger Education AAD innovation grant (LOI opens June 2025--5K/30K)

**Upcoming Events**:

1. May 7-10, 2025, San Diego, SID
2. July 10-13, 2025, Chicago, AAD Innovation Academy

**________________________________________________**

1. March 27-31, 2026 Denver, AAD
2. May 13-16, 2026 Chicago, SID

**Recommended:**

1. Brief Faculty Development Videos: <https://www.dermatologyprofessors.org/resources.php>
2. Listen to Dermasphere blog (Spotify)
